# School bullying as destructive communal coping of the school community

**DOI:** 10.3389/fpsyg.2022.1021765

**Published:** 2022-11-23

**Authors:** Alexandra A. Bochaver

**Affiliations:** Centre for Modern Childhood Studies, Institute of Education, National Research University Higher School of Economics, Moscow, Russia

**Keywords:** school bullying, theories of bullying, reasons of bullying, school stress, communal coping

## Introduction

Psychological theories suggest different explanatory models of school bullying and prescribe its likelihood, relying on the spectrum of the factors, from the individual predictors to the environmental ones which contribute to the increase or decrease of school bullying. This paper substantiates the questions about reasons and course of bullying and suggests a new conceptualization from the perspective of school community dynamics. A view of school bullying as a form of stress response, namely, the destructive communal coping of the school community, is proposed. This approach explains students' and teachers' joining bullying despite the values conflict and constancy of bullying.

The origin of bullying in different theories is explained in different ways. According to the social-cognitive approach, bullying perpetration is a result of a child's social learning, an adoption of the behavior which receives rewards and is typical for the social environment (Swearer et al., 2014). Bullying also is explained as a way for a bully to increase his/her popularity, visibility or to get other resources among the peers (Salmivalli, 2014). Another explanation of bullying suggests that it is determined by a desperate need to belong and can be a way of coping with a fundamental fear of social exclusion (Underwood and Ehrenreich, 2014). The bystanders' behavior (verbal or nonverbal acceptance of bullying; Salmivalli, 2010; Houghton et al., 2012) and moral disengagement (Hymel and Bonanno, 2014) promotes bullying, but does not trigger it by itself. The most influential social-ecological approach considers bullying as a phenomenon located in an extensive and complicated social context, including peer groups, schools, families, neighborhoods, communities, and country (Hong and Espelage, 2012; Hymel and Espelage, 2018). It allows an analysis of the risk and protection factors in relation to bullying in the various systems in which a child is socialized, and describes how the individual characteristics of children interacting with environmental contexts and systems prevent or support bullying (Espelage, 2014; Yoon and Bauman, 2014; Bauman et al., 2021). The ecological approach to bullying is is very helpful in conceptualizing separate groups of factors and bullying outcomes, however, it does not explain the reasons for the occurrence of bullying in general.

As P. Horton notes, ≪The problem with viewing school bullying through a macro lens is that by doing so, the social, institutional and societal contexts within which it occurs are left out of the picture≫ (Horton, 2016, p. 211). Reconstruction of possible causes of bullying shows that it performs a number of functions: it is a way of reproducing familiar and rewarding behavior; it helps to protect one's sense of belonging to a group; it establishes a social hierarchy and may provide the bully with power, popularity, and access to resources. However, there are questions that these theories cannot answer and highlight their insufficiency:

1. What motivates school students and even teachers to actively or passively support bullying, if they know that this is inappropriate behavior?
2. Why does the occurrence of bullying have such stability?

## Bullying as destructive communal coping

Despite the numerous anti-bullying programs developed in the last decades, there are a number of challenges. The average decrease in the prevalence of bullying is 15-20% or less (Gaffney et al., 2021). The programs do not work as efficiently and universally as planned; the teachers do not implement the interventions, and the adolescents do not react as expected; bullying returns to schools despite the programs (Cunningham et al., 2016; Nocentini et al., 2019; Salmivalli et al., 2021). These issues indicate that bullying is needed for something, it is a widely used and familiar tool for solving hidden social problems in different environments.

This paper suggests considering school bullying as a destructive form of communal coping (Afifi et al., 2020) with stress in the school community, and shows why this approach is promising in terms of reducing the problem.

Bullying as a coping strategy consists of (1) identifying several students as threatening the quality of the educational process or students' wellbeing, and (2) the subsequent direct or indirect displacement of them by the community majority to the position of marginal, alien, or rejected by the main group. This strategy allows the community to solve several problems: to reduce emotional tension by choosing a safe object for expressing aggression and emotional discharge; to establish a social hierarchy instead of uncertainty; to rally the remaining members of the collective around an artificially created confrontation; the latter is perhaps the most important. However, bullying has a high social price, due to the many negative consequences that affect children who participate in bullying, and therefore this strategy cannot be regarded as constructive.

### Stress

External events (education reforms, changes in legislation, social processes like war or epidemics), and internal ones (normative, like exams, or non-normative, like a change of leadership) can have a serious destructive impact on the school, forcing special efforts to maintain community integrity. The school interacts with the problematic situations, the solution to which may only be possible in joint activity within the framework of a holistic system. According to the concept of communal coping, people should perceive stress as co-experienced (Afifi et al., 2006). School bullying is not typical communal coping, so the concept of “Our stress, our responsibility” in this case is distorted. Apparently, there is a substitution: the original stressor remains hidden, and is replaced in the view of community members by an “identified stressor” (the behavior of a particular child or group of children). The association between stress and bullying prevalence may be caught in the evidence that bullying escalates before exams, with a change of teacher (Roland, 1999; Farmer and Xie, 2007), or after the transition from primary to secondary school (Salmivalli et al., 2021). Referring to G. Walton, Horton writes, that bullying often reflects larger social and political battles, moral panics, and collective anxieties (Horton, 2016).

### Shared coping strategy, synergy

School bullying has a complex role structure, it involves the interrelated activity of many school community members. The main task of individual coping is to adapt a person to the situational requirements, maintaining wellbeing, and reducing the effect of stress (Lazarus and Folkman, 1984), thus the fairly new concept of communal coping describes collective efforts to cope with a stressful event together (Lyons et al., 1998). Mutual assistance, the exchange of resources, information and emotional support helps to cope with some events more effectively, creating a sense of belonging and solidarity and reducing the experience of loneliness (Afifi et al., 2006). The paradox of bullying is that the community response causes a split by alienating the victim, but the process taking place around this fully meets the criteria above. Responding to an implicit stressor, the school community splits into a dominant privileged group and rejected participants, and a powerful energy is hidden in this confrontation. It is often perceived as justified by everyone except the victims, and rationalized explanations of bullying often contain xenophobic (nationalistic, homophobic, ableist, etc.) attitudes. Common pro-bullying narratives often support the idea that there is a fundamental difference between a child who has become a victim and others, and that the victim is responsible for bullying. Step by step, more and more people are involved in the bullying process. They join the victim-blaming narrative and the justification of the collective aggression. Collective moral disengagement happens: children and adults are actively involved in bullying or silently condone it, even if it is contrary to their values and is followed by shame and guilt. The group process seems to be more important in this case, than individual needs. The inefficiency of a zero-tolerance policy toward bullying, punishments, and bully exclusion (Boccanfuso and Kuhfeld, 2011; Bradshaw, 2013) confirms the communal character of bullying and its adaptive function.

### The destructiveness of bullying

Every coping strategy has certain benefits and costs (Lyons et al., 1998; Kuo, 2013). School bullying allows the most participants to join and to cope with stress emotionally in the short term, but it does not transform the underlying problem situation. There is a lot of evidence, that the victims, as well as the aggressors and the bystanders, face a number of serious negative consequences of bullying for their mental and physical health (e.g., anxiety and depressive symptoms, psychosomatic disorders, substance abuse, and self-harm), and social adjustment (e.g., problems with the close relationships, academic achievements, engagement in education, and stable employment), up to suicide (e.g., Copeland et al., 2013; Arseneault, 2018; Dhami et al., 2019). For the teachers, bullying may be a stressor which increase their burnout and exhaustion (Yoon and Bauman, 2014; Cunningham et al., 2016). All this points to the destructiveness of such a coping strategy in the long term.

### Recovery of the school community

Three clusters of school community recovery factors may be distinguished. First, *individual* factors (self-confidence, spirituality, maturity, positive attitudes of the community members, social and emotional learning): they make individuals more resilient, and their behavior becomes more prosocial (Divecha and Brackett, 2020). Second, *intra-school* factors (school climate, consistency of members' actions, cohesion and flexibility, openness in demanding and receiving support, collective narratives, posttraumatic growth; Chamlee-Wright and Storr, 2011; Wlodarczyk et al., 2016). As numerous bullying prevention programs and studies of their effectiveness show, bullying at school is reduced in terms of improving the quality and psychological safety of the environment as a whole and developing a systematic response to bullying situations from the school community (Divecha and Brackett, 2020; Dorio et al., 2020; Eldridge and Jenkins, 2020). Third, *extra-school* (economic and social resources, cooperation with other social institutions, community-based collaboration actions), by analogy with community recovery after natural disasters (Kusago, 2019).

## Discussion

Here are the answers to the research questions, based on the conception of school bullying as a form of destructive communal stress coping.

1. School students join bullying despite knowing that bullying is inappropriate behavior, because this is their contribution to the struggle with stress, uncertainty and emotional tension, and this goal becomes more important than their moral beliefs and attitudes. When teachers avoid discussing bullying, ignore children's victimization, or highlight favorite students, they also contribute to the collective struggle with stress, by joining bullying and receiving immediate behavioral support from the children.
2. The occurrence of bullying is stable because it has a number of social functions not explained only by the bully's individual level of aggression, and it reflects the more wide contexts. If the community lives with consistent stress and bullying matches its needs, bullying will return again and again despite any interventions which are implemented.

The proposed approach of considering bullying as a form of coping with stress by the whole school community opens up new opportunities for the development of anti-bullying interventions. They should begin with the acknowledgment of bullying as a community problem, and then include a number of transformations within the school and the involvement of a number of extra-school resources aimed at helping to reduce stress, restore community integrity and construct a new collective narrative. This approach seems to be a very complex and costly process, but it assumes the use of a “wide-angle lens” instead of a “macro lens” toward bullying, in the terms of Horton (2016), and gives hope to cope with the challenges faced by even the most effective anti-bullying programs (Salmivalli et al., 2021), due to a new framework that considers bullying not as an independent phenomenon, but as a consequence of a certain dynamics of the school community under stress.

## Author contributions

The author confirms being the sole contributor of this work and has approved it for publication.

## Conflict of interest

The author declares that the research was conducted in the absence of any commercial or financial relationships that could be construed as a potential conflict of interest.

## Publisher's note

All claims expressed in this article are solely those of the authors and do not necessarily represent those of their affiliated organizations, or those of the publisher, the editors and the reviewers. Any product that may be evaluated in this article, or claim that may be made by its manufacturer, is not guaranteed or endorsed by the publisher.
